# Supplementary material for: Organic carbon recycling in subduction zones
Source: Nat Commun. 2026 Apr 8;17:5000. doi: 10.1038/s41467-026-71559-w (PMC13237053; doi:10.1038/s41467-026-71559-w)
Supplement: Supplementary file 1 — Supplementary Information [file 41467_2026_71559_MOESM1_ESM.pdf]

Supplementary Information for
Organic carbon recycling in subduction zones

Baptiste Debret[1*], Pierre Bouilhol[2], Hélène Bouquerel[1], Thomas Rigaudier[2], Clément Herviou[1], Valentin Desmalles[1], Pierre-André Velut[1,3], Bénédicte Ménez[1], Stéphane Schwartz[4], Pierre Cartigny[1]

1: *Institut de physique du globe de Paris, Université Paris Cité, CNRS, Paris, France*
2: *Université de Lorraine, CNRS, CRPG, Nancy, France*
3: *Sorbonne Université, CNRS-INSU, ISTeP, Paris, France*
4: *Institut des Sciences de la Terre, Université Grenoble I, CNRS, Grenoble, France*
*Corresponding author: Baptiste Debret.

Email: debret@ipgp.fr

This PDF file includes:

Figures S1 to S6
Tables S1 to S5
SI References

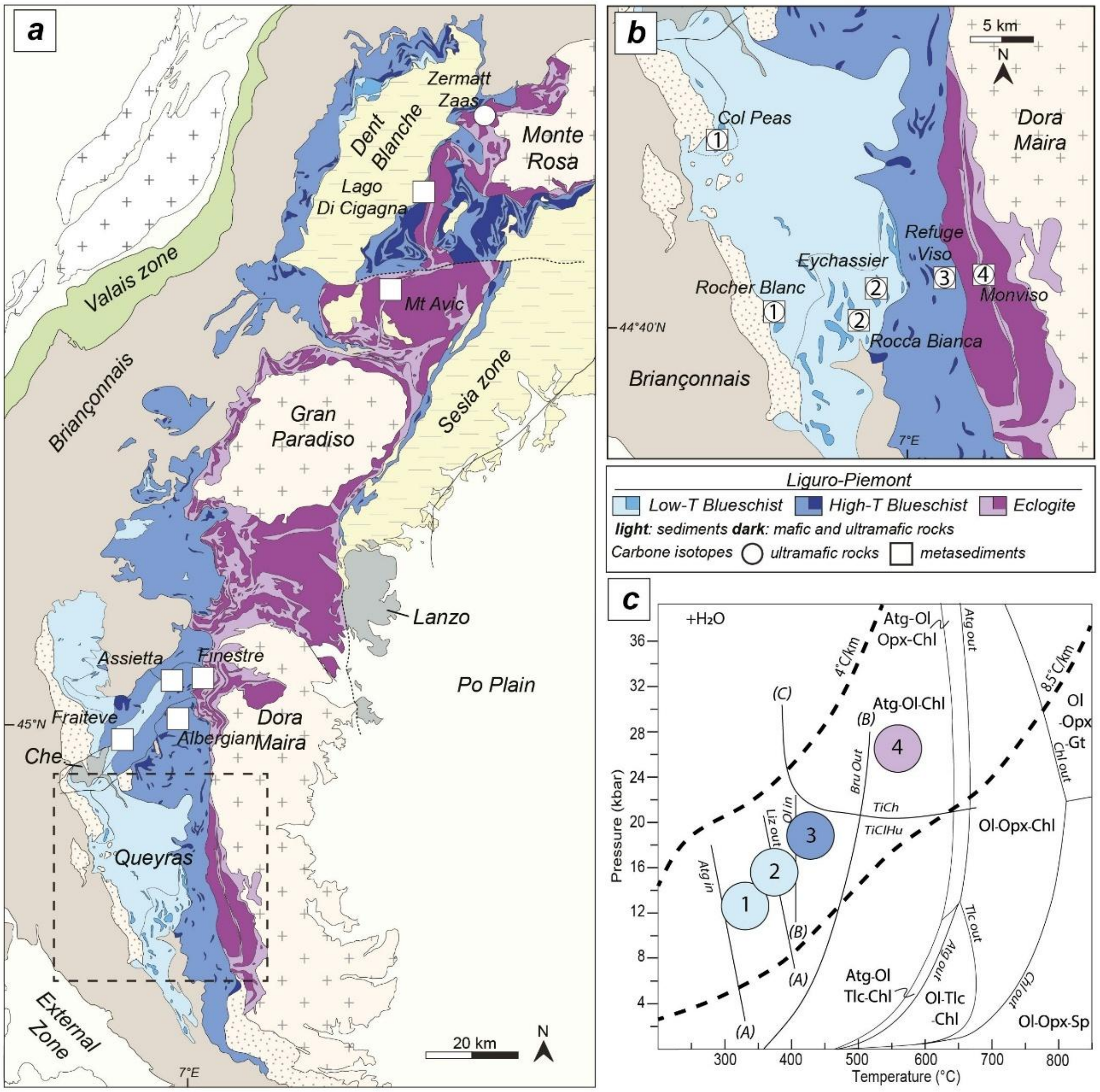

Fig. S1: Geological maps of the Western Alps and corresponding P-T record. (a) Simplified geological map of the Western Alps with the distribution of metamorphic facies within (modified from [1] with permission from Elsevier). The studied area is outlined by the black dashed square. Other localities where previous carbon isotope analyses were performed are reported (white squares). (b) Simplified geological map of the studied area. (c) P-T record of the studied metaophiolites. P-T estimates are from [2,3] and reference therein. Pressure–temperature estimates are primarily derived from thermodynamic modelling of associated metamafic rocks (metabasalts and metagabbros) and metasediments, complemented by Raman Spectroscopy of Carbonaceous Material (RSCM) thermometry applied to metasediments. Because direct quantitative P–T determinations in metaserpentinites are intrinsically difficult, the P–T record of serpentinites is considered semi-quantitative. In metaserpentinites, constraints are mainly based on well-established phase transitions that are predominantly temperature-dependent. These include: (A) lizardite–antigorite transition at ~300–400 °C [4], (B) brucite breakdown at ~450–550 °C [5], and (C) titanoclinohumite (TiClHu) –titanochondrodite (TiCh) transition at pressures of ~2.2–2.5 GPa [6]. Together, these transitions define broad P–T fields rather than discrete conditions, which are

sufficient for placing the formation and preservation of abiotic organic carbon within the prograde metamorphic evolution of subducting serpentinites.

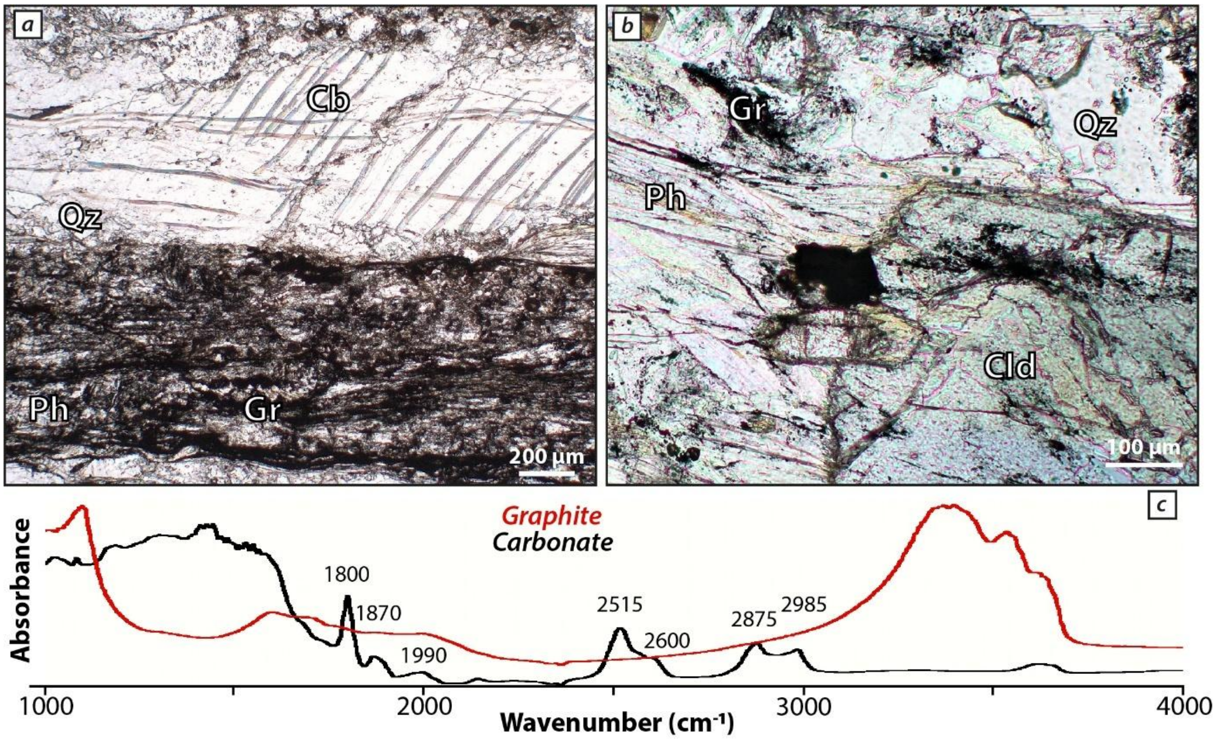

Fig. S2: Characterization of carbonate and organic matter in metasediments across the Queyras-Monviso transect. (a) Thin section observation of a Queyras metasediment mainly made of alternating layers of carbonate and organic matter associated with quartz, phengite and garnet boundaries. (b) Thin section observation of organic matter associated with quartz, phengite and chloritoid boundaries in the Monviso massif. (c) Examples of raw FTIR spectra of carbonate and organic matter in the metasediments of the Monviso massif. While carbonate display intense bands at 2515, 2600, 2875 and 2985 cm^{-1}, the graphite does not show any bands associated with carbon bounds at high frequency, only silicates and OH bounding were detected and attributed to the silicate matrix associated with organic matter.

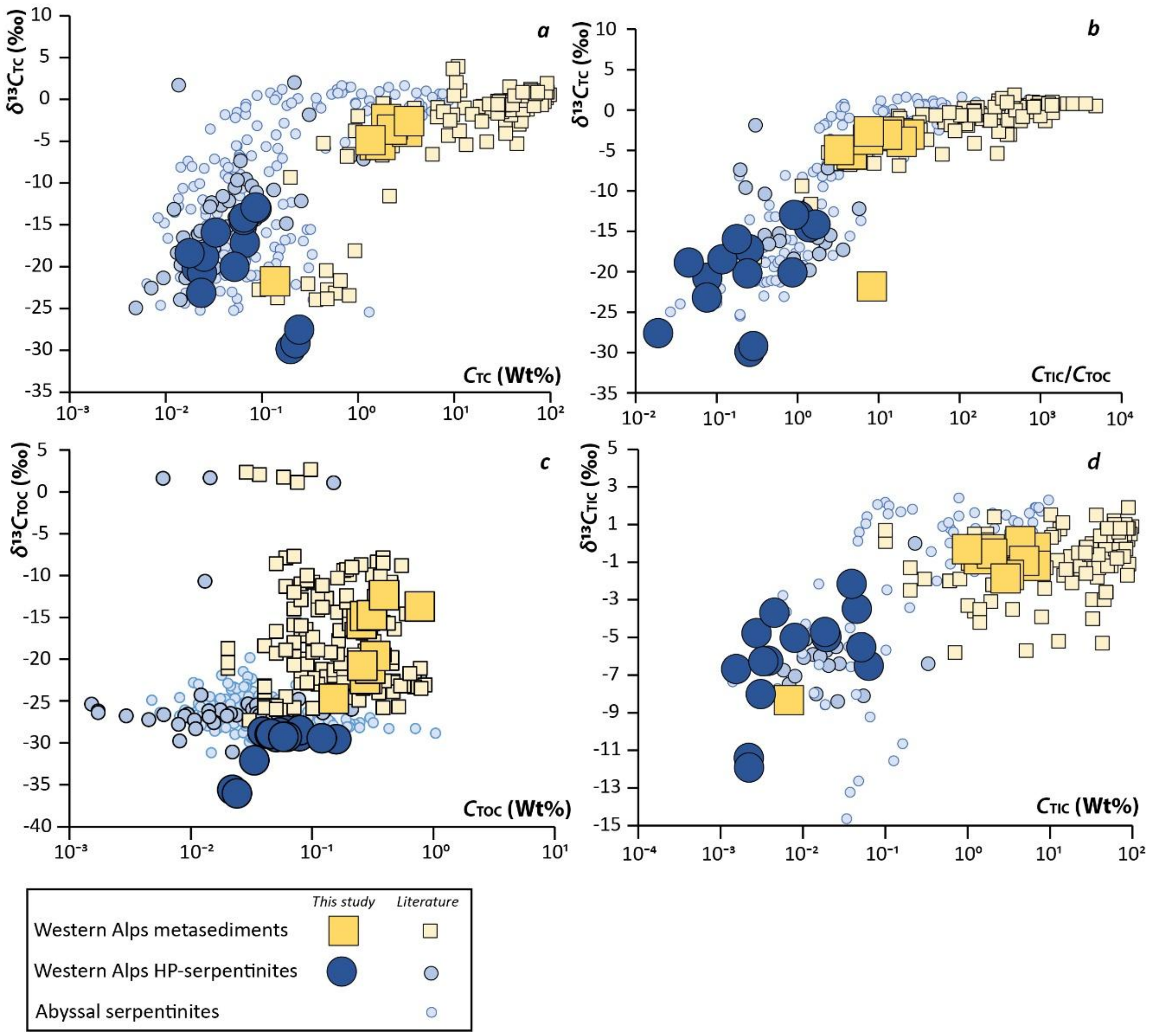

Fig. S3: Bulk rock carbon (total carbon, TC; total organic carbon, TOC; total inorganic carbon, TIC) concentrations and isotope ($\delta^{13}C$) compositions of the studied samples compared with previous analyses in the Western Alps and abyssal serpentinites**.** (a) Plot of $\delta^{13}C_{TC}$ vs C_{TC}. (b) Plot of $\delta^{13}C_{TC}$ vs C_{TIC}/C_{TOC} ratio. (c) Plot of $\delta^{13}C_{TOC}$ vs C_{TOC}. (d) Plot of $\delta^{13}C_{TIC}$ vs C_{TIC}. Note that the high C_{TIC} in abyssal serpentinites mainly correspond to ophicarbonates sampled near the surface while with depth the CTIC quickly decreases [7]. The abyssal data set is from [8]. The Western Alps dataset includes rocks from the Queyras Schiste Lustrès complex (this study and [9]), Syros [10], Cerro del Almirez [11], Zermatt Zaas [12], Monviso (this study and [9]) and Cima Di Gagnone [13] metaophiolites. Please refer to method section for the error bars.

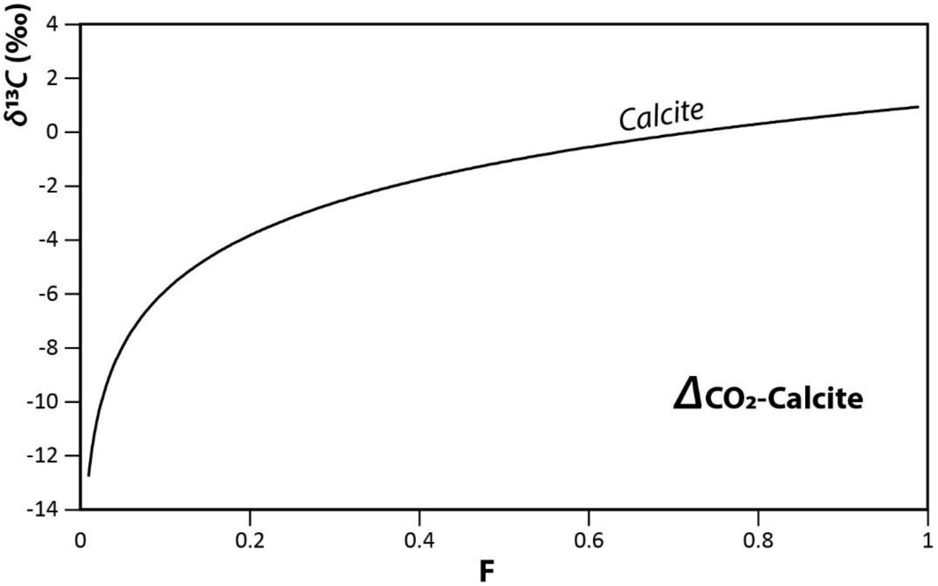

Fig. S4: Changes in $\delta^{13}C$ values of carbonate during devolatilization with a ΔCO2-Calcite of 3 [14]. The Rayleigh distillation model is calculated according to [15] δf – δi = 1000 ($F^{\alpha-1}$-1), F is the fraction of carbon remaining, α the fractionation factor (fluid-rock) and δf, δi the final and initial isotopic composition of carbonate (with δi = 1‰).

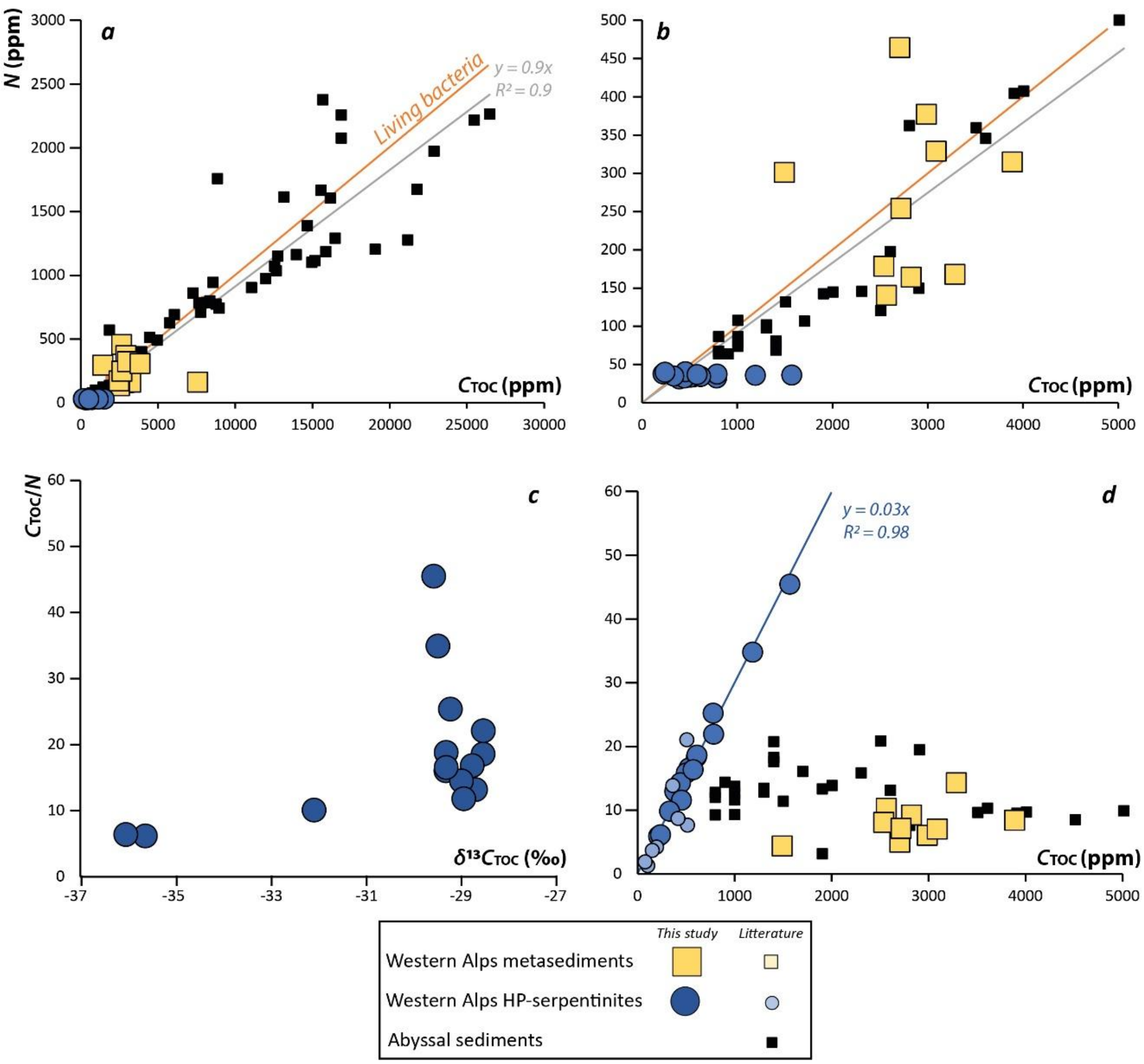

Fig. S5: Variations of nitrogen concentrations between metaserpentinites and metasedimentary rocks. (a-b) Plots of N vs C_{TOC}. The living bacteria ratio is from [16]. (c) Plot of C_{TOC}/N ratio vs $\delta^{13}C_{TOC}$. (d) Plot of C_{TOC}/N ratio vs C_{TOC}. Abyssal sediment dataset is from [17]. The Western Alps dataset includes rocks from the Queyras Schistes Lustrés complex, Monviso (this study) and Cima Di Gagnone [13] metaophiolites. Please refer to method section for the error bars.

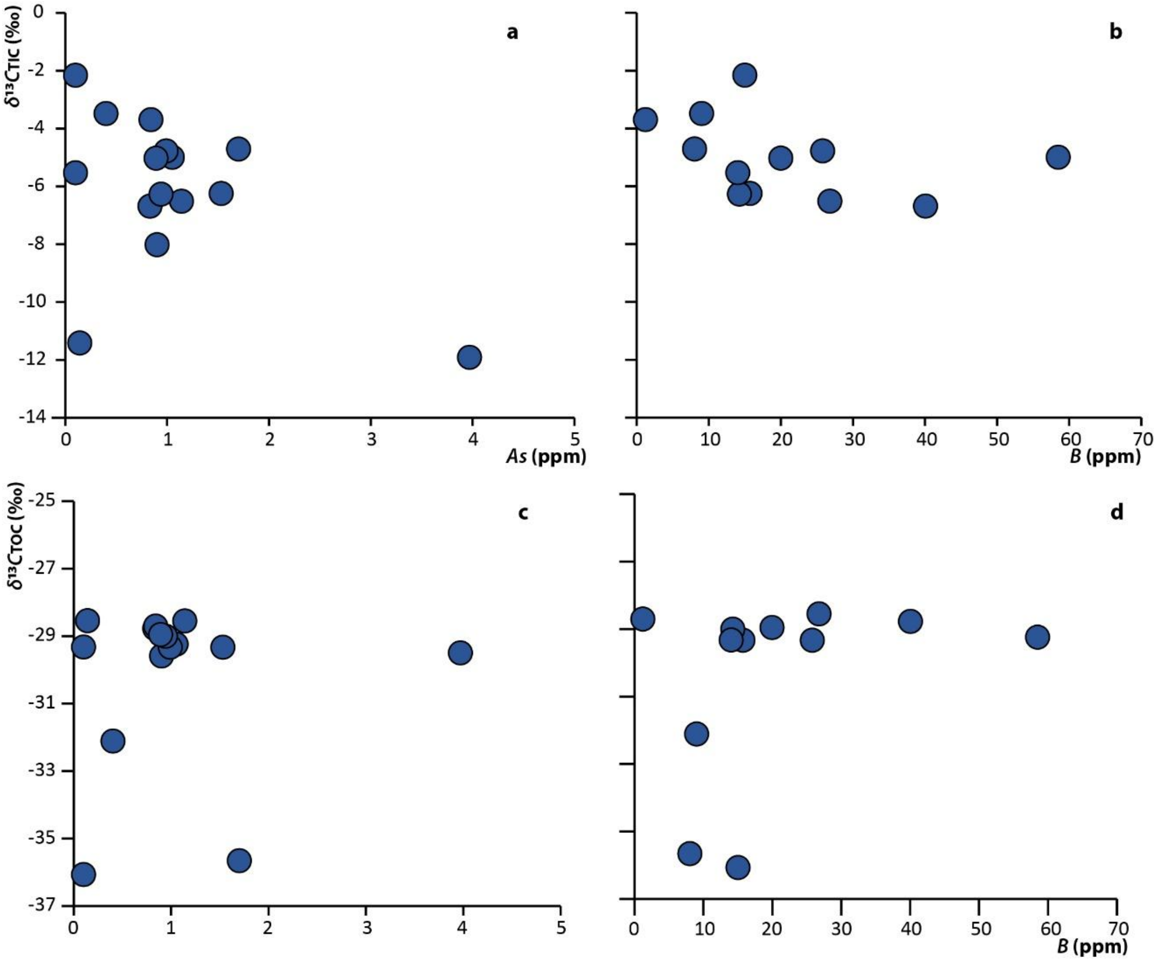

Fig. S6: Variations of $\delta^{13}C_{TIC}$ (a-b) and $\delta^{13}C_{TOC}$ (c-d) vs fluid mobile element (FME) concentrations in metaserpentinites. Enrichments in FMEs such as As and B are characteristic of open-system addition of sedimentary-derived components [18]. The lack of systematic correlation with $\delta^{13}C$ indicates that the observed isotopic fractionations are not controlled by sediment input or open-system organic precipitation.

Table S1. Locations and main silicate assemblages composing the studied samples.

Metamorphic Unit	Sample Name	Locality	Lithology	Lat.	Long.	Main Silicate mineralogy
ULP	CP1	Col Peas	Metasediment	44.815	6.804	Lws, Car, Qz, Ph, Chl
	CP2	Col Peas	Metaserpentinite	44.815	6.804	Atg, Di, Chl, Mt
	CP3	Col Peas	Metaserpentinite	44.815	6.804	Atg,± Chl, Mt
	CP8	Col Peas	Metaserpentinite	44.815	6.804	Liz, Atg, ±Chl
	RQ36	Rocher Blanc	Metasediment	44.680	6.870	Lws, Car, Qz, Ph, Chl
	RQ30	Rocher Blanc	Metaserpentinite	44.680	6.870	Liz, Mt
	RQ26	Rocca Bianca	Metasediment	44.675	3.991	Lws, Car, Qz, Ph, Chl, ±Cld
	RQ28	Rocca Bianca	Metasediment	44.675	3.991	Lws, Car, Qz, Ph, Chl, ±Cld
	RQ23	Rocca Bianca	Metaserpentinite	44.675	3.991	Liz, Atg, Mt
	RQ22	Eychassier	Metasediment	44.739	6.968	Lws, Car, Qz, Ph, Chl, ±Cld
	CE3	Eychassier	Metaserpentinite	44.739	6.968	Atg, Chl, Mt
	CE10	Eychassier	Metaserpentinite	44.739	6.968	Atg, Liz, ±Di
	CE14b	Eychassier	Metaserpentinite	44.739	6.968	Di, Chl ±Atg
MLP	RQ04	Refuge du Viso	Metasediment	44.701	7.100	Cld, Lws, Qz, Ph, Chl
	RQ08	Refuge du Viso	Metasediment	44.701	7.100	Cld, Lws, Qz, Ph, Chl
	RV5	Refuge du Viso	Metasediment	44.701	7.100	Cld, Lws, Qz, Ph, Chl
	RV3	Refuge du Viso	Metaserpentinite	44.701	7.100	Atg, Ol2, Mt
	RV8	Refuge du Viso	Metaserpentinite	44.701	7.100	Atg, ±Liz, Mt
	RQ01	Refuge du Viso	Metaserpentinite	44.701	7.100	Atg, Mt
LLP	V11	Monviso	Metasediment	44.694	7.155	Cld, Qz, Ph
	V15	Monviso	Metasediment	44.694	7.155	Cld, Qz, Ph
	Vis1813	Monviso	Metasediment	44.701	7.085	Qz, Ph, Gr
	Vis1817	Monviso	Metasediment	44.669	7.121	Qz, Ph, Gr
	Vis2011	Monviso	Metaserpentinite	44.747	7.075	Atg, Ol2, Mt, Di
	Vis2014	Monviso	Metaserpentinite	44.747	7.075	Atg, Ol2, Chl, Brc, Mt, TiCl
	Vis185A2	Monviso	Metaserpentinite	44.716	7.087	Atg, Ol2, Mt, TiCl/TiChn, Brc, Di1, Chl
	Vis184	Monviso	Metaserpentinite	44.716	7.087	Atg, Ol2, Mt, TiCl/TiChn, Brc, Di1, Chl

U/M/L LP: Upper/Middle/Lower Liguro Piemont Unit; Lws: Lawsonite, Car: Carpholite, Gr: Garnet; Qz: Quartz, Ph: Phengite, Chl: Chlorite, Atg: Antigorite, Mt: Magnetite, Liz: Lizardite, Cld: Chloritoide, Ol2: Metamorphic Olivine, Brc: Brucite; TiCl/TiChn: titanoclinohumite–titanochondrodite

Table S2. Nitrogen and carbon concentrations and isotopic compositions of the standards run alongside samples during the C_{TC} and C_{TOC} analytical sessions.

		**[*N*] ppm**	**[C_{TC}] wt%**	**$\delta^{13}C_{TC}$**
Standards for Concentrations				
AGV-1	mean	42	0.018	
n=13	2s	12	0.010	
BFSd	mean	580	0.52	-21.5
n=40	2s	84	0.04	1.1
CRPG_M2	mean	697	0.40	-25.3
n=24	2s	61	0.02	1.8
EM_B2153	mean	1351	1.91	
n=8	2s	316	0.22	
EM_B2189	mean	193	0.25	
n=12	2s	51	0.02	
SOIL MIX_4	mean	484	2.36	
n=11	2s	140	0.01	
Standards for Isotopic Compositions				
BFSd	mean	580	0.52	-21.5
n=40	2s	84	0.04	1.1
CRPG_M2	mean	697	0.40	-25.3
n=24	2s	61	0.02	1.8
IAEA 600	mean			-27.9
n=13	2s			0.6
IAEA CH6	mean			-10.7
n=6	2s			0.6
NBS22	mean			-30.2
n=5	2s			0.4
USGS24	mean			-16.4
n=5	2s			0.4
IAEA_CH7	mean			-31.9
n=2	2s			0.02
UB-N		50	0.06	-14.0
UB-N		38	0.06	-13.7

Table S3. N concentrations, C_{TOC} and $\delta^{13}C_{TOC}$ of uncombusted and precombusted samples.

Sample Name	Precombustion	[C_{TOC}]	$\delta^{13}C_{TOC}$	N
V15	None	0.28	-16.1	337
	400°C	0.23	-14.1	311
Vis1813	None	0.30	-15.2	422
	400°C	0.28	-14.9	422
Vis1817	None	0.39	-13.3	446
	400°C	0.37	-11.8	467
Vis185A2	None	0.03	-35.9	44
	400°C	0.02	-36.2	46
Vis2011	None	0.03	-33.9	46
	400°C	0.02	-33.4	43
Vis2014	None	0.03	-35.6	48
	400°C	0.02	-35.7	44

Table S4 Carbon concentrations and isotopic compositions of the standards run alongside samples during the C_{TIC} analytical sessions

		[C_{TIC}] wt%	$\delta^{13}C_{TIC}$
BR516	mean	0.35	-1.2
n=11	2s	0.02	0.1
BR8107	mean	0.65	-1.1
n=11	2s	0.03	0.1
CA10-08	mean	1.35	-1.8
n=2	2s	0.08	0.1
NAG7RT	mean	2.01	-1.1
n=2	2s	0.16	0.1
SARM CAL-S_2003	mean	12.07	2.7
n=8	2s	0.32	0.1
UBN	mean	0.03	-7.0
n=12	2s	0.01	1.6

Table S5. Nitrogen, carbon concentrations and isotope ratios in the studied samples (M: metasediments, S: metaserpentinite).

	[*N*] ppm	[C_{TC}] wt%	$\delta^{13}C_{TC}$	[C_{TOC}]	$\delta^{13}C_{TOC}$	[C_{TIC}]	$\delta^{13}C_{TIC}$
Col Peas							
CP1 (M)	162	2.71	-3.1	0.28	-22.3	2.47	-0.2
CP2 (S)	33	0.08	-13.1	0.06	-28.5	0.06	-6.6
						0.06	-6.4
CP3 (S)	31	0.06	-17.2	0.08	-29.2	0.02	-5.0
						0.02	-5.0
CP8 (S)	32	0.05	-20.2	0.05	-28.8	<0.01	-7.1
						<0.01	-6.2
Rocher Blanc							
RQ36 (M)	300	0.13	-21.8	0.15	-24.7	0.01	-8.3
						0.01	-8.4
RQ30 (S)	36	0.19	-29.9	0.08	-28.5	<0.01	-11.3
						<0.01	-11.5
Rocca Bianca							
RQ26 (M)	463	1.44	-5.5	0.27	-22.4	1.22	-0.6
				0.28	-21.9	1.30	-0.7
RQ28 (M)	393	1.67	-6.5	0.31	-20.4	1.37	-0.9
	358	1.64	-4.5	0.29	-21.9	1.44	-0.9
RQ23 (S)	34	0.22	-29.2	0.16	-29.6	<0.01	-8.0
						<0.01	-8.0
Eychassier							
RQ22 (M)	166	2.57	-2.7	0.33	-19.7	2.16	0.2
						2.16	0.0
CE3 (S)	31	0.05	-20.8	0.05	-29.3	<0.01	-6.3
						<0.01	-6.2
CE10 (S)	33	0.06	-18.9	0.06	-29.3	<0.01	-4.9
						<0.01	-4.7
CE14b (S)	30	0.04	-18.5	0.04	-28.7	<0.01	-3.6
						<0.01	-3.8
Refuge du Viso							
RQ04 (M)	167	2.74	-3.2	0.76	-13.8	2.52	-1.2
				0.76	-13.6		
RQ08 (M)	139	3.13	-3.9	0.23	-20.3	2.17	-0.9
				0.28	-21.3	2.27	-1.0
RV5 (M)	177	2.21	-3.1	0.25	-15.7	1.95	-1.8
				0.26	-15.9	1.90	-1.6
RV3 (S)	31	0.05	-23.2	0.04	-29.0	<0.01	-6.2
						<0.01	-6.4
RV8(S)	39	0.05	-16.0	0.05	-29.0	0.01	-5.2
						0.01	-4.9
RQ01 (S)	34	0.24	-27.6	0.12	-29.5	<0.01	-11.6
						<0.01	-12.3

Monviso							
V11 (M)	125	1.84	-3.1	n.d.	n.d.	1.84	-0.3
	142	1.94	-1.7	n.d.	n.d.	1.87	-0.4
V15 (M)	242	2.10	-4.9	0.31	-14.4	2.02	-0.6
	263	2.24	-2.5	0.28	-16.1	1.95	-0.5
				0.23	-14.1		
Vis1813 (M)	344	1.31	-5.6	0.34	-14.2	0.96	-0.3
	311	1.29	-4.3	0.30	-15.2	1.00	-0.3
				0.28	-14.9		
Vis1817 (M)	313	3.27	-2.7	0.40	-12.0	1.83	-1.9
				0.39	-13.3	1.83	-1.9
				0.37	-11.8		
Vis2011 (S)	33	0.06	n.d.	0.06	-28.8	0.05	-3.5
	33	0.06	-14.5	0.03	-33.9	0.04	-3.5
				0.02	-33.4		
Vis2014 (S)	36	0.05	-20.1	0.03	-35.6	0.02	-4.6
				0.02	-35.7	0.02	-4.8
Vis185A2 (S)	38	0.06	-14.2	0.03	-35.9	0.04	-2.2
				0.02	-36.2	0.04	-2.1
Vis184 (S)	35	0.08	-13.0	0.06	-29.3	0.06	-5.6
						0.04	-5.4

n.d. not detected

Table S6. Summary Table Showing the influx characteristic of organic and total carbon in metasediments for individual subduction zone. C_{TOC}/C_{TC} and Total Carbon Subducted data are from [19], Carbonate recycling efficiency is from

Trench	C_{TOC}/C_{TC} (%)	Total Carbon Subducted (Mt/yr)	Predicted Subducted $\delta^{13}C$ (‰)	Carbonate recycling efficiency (%)	Total Carbon Recycled (Mt/yr)	Carbon recycling efficiency (T/yr/km)	HP carbonate $\delta^{13}C$ (‰)	Predicted Recycled $\delta^{13}C$ (‰)
N. Chile	16.8	3.28	-2.86	0.7	2.46	1231	-0.07	-3.76
Peru	16.7	3.17	-2.8	0.54	1.96	889	-0.85	-4.38
Colombia-Ecuador	13.5	2	-2.1	0.42	1.00	906	-1.60	-4.36
Costa Rica	12	0.69	-1.8	0.4	0.33	724	-1.75	-4.18
Nicaragua	12.9	0.31	-2	0.4	0.15	538	-1.75	-4.36
Guatamala	10.1	1.5	-1.3	0.35	0.62	1247	-2.15	-4.15
Mexico	62.8	0.4	-13.4	0.68	0.35	207	-0.16	-13.87
Kuril	73.3	0.2	-15.9	0.32	0.16	149	-2.42	-16.77
Kamchatka	14.4	1.54	-2.3	0.22	0.51	465	-3.54	-6.20
NE Japan	89.2	0.44	-19.5	0.38	0.41	411	-1.90	-19.83
Mariana	4.9	0.72	-0.1	0.22	0.19	116	-3.54	-4.44
Izu-Bonin	89.4	0.06	-19.6	0.28	0.06	43	-2.82	-19.97
Ryukyu	59.5	0.02	-12.7	0.6	0.02	17	-0.53	-13.31
South Luzon	1.1	1.72	0.8	0.6*	1.04	2599	-0.53	-0.77
Philippine-Mindanao	0.8	1.3	0.8	0.38	0.50	500	-1.90	-2.06
Tonga	26.5	0.34	-5.1	0.22	0.15	97	-3.54	-8.43
Kermadec	7.7	2.17	-0.8	0.18	0.53	422	-4.14	-5.51
Solomons	5.1	4.8	-0.2	0.38	1.98	718	-1.90	-2.93
South Sandwich	31.9	0.27	-6.3	0.6*	0.20	281	-0.53	-7.38
Southern Chile	49.6	0.66	-10.4	0.92	0.63	317	0.75	-10.53
Lesser Antilles	21.4	1.29	-3.9	0.3	0.58	683	-2.61	-6.76
Oregon-Washington	28.7	0.91	-5.6	0.6*	0.65	765	-0.53	-6.69
Cascadia British Columbia	56.5	0.44	-12	0.14	0.28	501	-4.89	-14.56
Aleutians North Pacific	38.3	1.76	-7.8	0.52	1.24	826	-0.96	-9.02
Alaska	62.5	2.76	-13.4	0.68	2.43	1185	-0.16	-13.81
Taiwan-North Luzon	30	0.74	-5.9	0.6*	0.53	761	-0.53	-6.97
SW Japan	40.7	1	-8.4	0.58	0.75	834	-0.64	-9.33
Sumatra	6.5	6.66	-0.5	0.3	2.30	2557	-2.61	-3.87
Java	6.5	4.29	-0.5	0.1	0.68	324	-5.89	-6.94
Andaman-Burma	17.4	7.73	-3	0.6*	5.18	2876	-0.53	-4.27
Makran	16.9	4.71	-2.9	0.6*	3.14	3144	-0.53	-4.16
Aegean-Mediterranean	11.4	1.76	-1.6	0.38	0.79	661	-1.90	-4.19

*Average value as no individual data was available

Supplementary References

1. Herviou, C. *et al.* Subducted fragments of the Liguro-Piemont ocean, Western Alps: Spatial correlations and offscraping mechanisms during subduction. *Tectonophysics* **827**, 229267 (2022).
2. Herviou, C. *et al.* Fractal distribution of subduction-related crack-seal veins (Schistes Lustrés, W. Alps): Implications for fluid flow and rupture processes at the downdip end of the seismogenic zone. *J. Geophys. Res. Solid Earth* **128**, e2022JB026317 (2023).
3. Agard, P. Subduction of oceanic lithosphere in the Alps: Selective and archetypal from (slow-spreading) oceans. *Earth-Science Rev.* **214**, 103517 (2021).
4. Schwartz, S. *et al.* Pressure-temperature estimates of the lizardite/antigorite transition in high pressure serpentinites. *Lithos* **178**, 197–210 (2013).
5. Caurant, C., Debret, B., Ménez, B., Nicollet, C. & Bouilhol, P. Redox heterogeneities in a subducting slab: Example from the Monviso meta-ophiolite (Western Alps, Italy). *Lithos* 107136 (2023) doi:https://doi.org/10.1016/j.lithos.2023.107136.
6. Shen, T., Hermann, J., Zhang, L., Padrón-Navarta, J. A. & Chen, J. FTIR spectroscopy of Ti-chondrodite, Ti-clinohumite, and olivine in deeply subducted serpentinites and implications for the deep water cycle. *Contrib. to Mineral. Petrol.* **167**, 992 (2014).
7. Schwarzenbach, E. M., Früh-Green, G. L., Bernasconi, S. M., Alt, J. C. & Plas, A. Serpentinization and carbon sequestration: A study of two ancient peridotite-hosted hydrothermal systems. *Chem. Geol.* **351**, 115–133 (2013).
8. Debret, B., Andreani, M. & Godard, M. A review of abyssal serpentinite geochemistry and geodynamics. *Earth-Science Rev.* **258**, 104910 (2024).
9. Cook-Kollars, J., Bebout, G. E., Collins, N. C., Angiboust, S. & Agard, P. Subduction zone metamorphic pathway for deep carbon cycling: I. Evidence from HP/UHP metasedimentary rocks, Italian Alps. *Chem. Geol.* **386**, 31–48 (2014).
10. Schwarzenbach, E. M. *et al.* Sulphur and carbon cycling in the subduction zone mélange. *Sci. Rep.* 1–11 (2018) doi:10.1038/s41598-018-33610-9.
11. Alt, J. C. *et al.* The role of serpentinites in cycling of carbon and sulfur : Seafloor serpentinization and subduction metamorphism. *Lithos* **178**, 40–54 (2013).
12. Bouilhol, P. *et al.* Decoupling of inorganic and organic carbon during slab mantle devolatilisation. *Nat. Commun.* **13**, 308 (2022).
13. Cannaò, E., Tiepolo, M., Bebout, G. E. & Scambelluri, M. Into the deep and beyond : Carbon and nitrogen subduction recycling in secondary peridotites. *Earth Planet. Sci. Lett.* **543**, 116328 (2020).
14. Chacko, T., Mayeda, T. K., Clayton, R. N. & Goldsmith, J. R. Oxygen and carbon isotope fractionations between CO2 and calcite. *Geochim. Cosmochim. Acta* **55**, 2867–2882 (1991).
15. Valley, J. W. Stable isotope geochemistry of metamorphic rocks. *Rev. Mineral. geochemistry* **16**, 445–489 (1986).
16. Ader, M. *et al.* Nitrogen isotopic evolution of carbonaceous matter during metamorphism: Methodology and preliminary results. *Chem. Geol.* **232**, 152–169 (2006).
17. Li, L. & Bebout, G. E. Carbon and nitrogen geochemistry of sediments in the Central American convergent margin: Insights regarding subduction input fluxes, diagenesis, and paleoproductivity. *J. Geophys. Res. Solid Earth* **110**, (2005).
18. Lafay, R. *et al.* High-pressure serpentinites, a trap-and-release system controlled by metamorphic conditions: Example from the Piedmont zone of the western Alps. *Chem. Geol.* **343**, (2013).
19. Clift, P. D. A revised budget for Cenozoic sedimentary carbon subduction. *Rev. Geophys.* **55**, 97–125 (2017).
